# Framing anticoagulation control according to clinical practice for patients with atrial fibrillation in Spain: a multidisciplinary vision trough AMFA Project

**DOI:** 10.3389/fcvm.2025.1426072

**Published:** 2025-03-05

**Authors:** Juan Cosín-Sales, Jose Polo-García, Olga Gavín Sebastián, Marta Rubio Cabezas, María Lloret Avellá

**Affiliations:** ^1^ Department of Cardiology, Hospital Arnau de Vilanova, Valencia, Spain; ^2^ Primary Care, Centro de Salud de Casar de Cáceres, Cáceres, Spain; ^3^ Department of Hemathology, Hospital Clínico Universitario Lozano Blesa, Zaragoza, Spain; ^4^ Real World Evidence (RWE), IQVIA S.A, Madrid, Spain

**Keywords:** atrial fibrillation, anticoagulation, time in therapeutic range (TTR), international normalized ratio (INR), vitamin k antagonists (VKA), direct oral anticoagulants (DOAC)

## Abstract

**Introduction and objectives:**

The use of vitamin K antagonists (VKA) in patients with atrial fibrillation (AF) in Spain remains high, even though the available data on anticoagulation control (TRT, time in therapeutic range) shows suboptimal results. The objective of the AMFA project, an atlas of the management of anticoagulation in patients with AF, was to describe oral anticoagulation management in Spanish´ patients.

**Materials and methods:**

The AMFA Project is a descriptive, multicenter study. It included information from 60 healthcare areas from each of the 17 Spanish regions. Consensus methodologies were used to analyze qualitative information obtained from the physicians' experience and perception and quantitative data collected through a specialized study questionnaire. In this questionnaire, experts were asked to include data of the last 10 consecutive patients attended with AF on anticoagulation treatment.

**Results:**

Records from 1,580 patients were obtained from 176 experts. Of them, 34.7% were cardiologists, 32.9% general practitioners (GPs), and 32.4% hematologists. The utilization rates of Direct Oral Anticoagulants (DOACs) and VKAs in clinical practice was 55.8% and 43.3%, respectively, which was not correlated with experts' perception. Clinical practice data revealed that 30.3% of the patients included did not have international normalized ratio (INR) or TTR data available, while only 3.8% of the experts reported that INR/TTR information was not available according to their perception. Considering only patients who had INR and TTR available, clinical practice showed that 59.0% of the patients had their coagulation in range, while the remaining 41.0% were uncontrolled. This result matches with the general perception reported by the experts, 62.6% of patients in range. Additionally, up to 22.2% of patients received DOAC treatment at suboptimal doses.

**Conclusions:**

These data highlight the suboptimal control of the INR of patients, as well as the difficulties in DOACs access in Spain. The study uncovers the need to implement actions to improve INR control, facilitate access to DOACs treatment, and standardize AF patients' management. Establishing protocols that facilitate intervention may optimize the management of the patients with AF.

## 1 Introduction

Atrial fibrillation (AF) is the most common chronic cardiac arrhythmia. The prevalence of AF in Spain is >4% in the population over 40 years, rising with increasing age older than 60 years, and 17.7% among those over 80 years (1, 2). AF is associated with high morbidity and mortality due to an increased risk of thromboembolic events and other cardiac complications. The most severe consequence of AF is the risk of stroke (3, 4).

Effective management of AF involves anticoagulation, cardiac rhythm and rate control (5). In Spain, the most common anticoagulant therapy are vitamin K antagonists (VKAs) (6). However, VKAs present significant limitations such as a narrow therapeutic range, numerous drug/food interactions, and the need of frequent monitoring and dose adjustments (7, 8). VKAs dosing is monitored through the calculation of the international normalized ratio (INR). The quality of the anticoagulation treatment is determined though the calculation of the time in therapeutic range (TTR). Adequate anticoagulation control is achieved when TTR is >60%–65%. Therefore, a patient is considered to have poorly controlled coagulation when their TTR falls below 60%–65% (9).

DOACs have shown a similar or even more favorable efficacy and safety profile compared to VKAs (10), and are considered first-line therapy for the prevention of stroke and systemic embolism in patients with AF (5). However, in Spain DOACs prescription requires an inspection visa for reimbursement and is often reserved for patients whom VKA treatment fails to maintain an adequate TTR (11). This can lead to delays in treatment access, inconveniences for patients, variability in access conditions among regions (12), and lower use of DOACs in Spain (60.2%) compared to other European countries (78%–92%) (13, 14).

Evidence suggests that only 40%–50% of the patients treated with VKAs achieve an INR within the therapeutic range at least 65% of the time. This may lead to suboptimal anticoagulation and an increased risk of serious events for AF patients (7, 15, 16).

Several scientific societies and healthcare settings have established guidelines to facilitate effective anticoagulation management in AF patients (17, 18). Nevertheless, recognized discrepancies among healthcare professionals underscore the need for standardized protocols (19).

The objective of this study is to understand the current status of anticoagulation healthcare in Spain and to identify best practices. Their implementation across different centers and health areas could optimize the management of anticoagulated patients.

## 2 Materials and methods

### 2.1 Study design

The Atlas of Anticoagulation Management in Patients with Atrial Fibrillation, the AMFA Project, is a descriptive, multicenter study conducted in Spain. It included information of 60 different healthcare areas belonging to each of the 17 Spanish regions. It was endorsed and coordinated by three Scientific Societies: Spanish Society of Cardiology (SEC), Spanish Society of Primary Care Physicians (SEMERGEN), and Spanish Society of Thrombosis and Hemostasis (SETH). AMFA Project design, study materials, and the selection of the sanitary areas, participants, and final outcomes, were validated by a scientific committee that includes three experts from each scientific society.

The study relies on consensus-based methodologies that combine qualitative information obtained from the participating physicians experience and perception and quantitative data collected through a study specific questionnaire. In this questionnaire, experts were asked to include data of the last 10 consecutive patients with AF on anticoagulation treatment attended. The study included 60 meetings between April 2022 and May 2023.

Specific criteria for the completion of the questionnaire and group meeting dynamics were focused on patients diagnosed with nonvalvular AF who initiated oral anticoagulant treatment. Center care protocols of each participant and routine clinical practice were considered.

### 2.2 Objectives of the study

The primary objective of AMFA study was to comprehensively analyze the implementation of oral anticoagulation management in Spain across multidisciplinary teams and regional areas and identify best practices and areas for enhancing patient treatment. Thus, the percentage of patients treated with VKAs and TTR ≥60% was quantified and compared against physicians' perception about this control.

Secondary objectives were to describe the AF patient journey for anticoagulation management, identify guidelines and protocols used in clinical practice, monitor long-term anticoagulated patients, assess INR control, identify weaknesses and strengths in patient management, and determine the decision-making process to switch patients from VKAs to DOACs, and the DOACs doses used in clinical practice.

### 2.3 Study population

Two different populations participated in the AMFA project. Medical professionals across 60 healthcare areas in Spain specialized in Cardiology, Hematology, and Primary Care (PC) and patients with AF. Patients with moderate or severe rheumatic mitral stenosis and/or mechanical cardiac valvular prostheses were excluded.

Sample size for the experts was calculated considering that, with 169 physicians interviewed and having responded INR and TRT-related items, we would obtain a marginal error of approximately 7.5% for the primary objective, considering below 10% acceptable. With data from 843 patients having INR and TRT responses, a precision of at least 5% would be expected for patient-related categorical evaluations, assuming an expected proportion of 50% (thus, assuming maximum indetermination), and a 95% level of confidence.

### 2.4 Ethical considerations

As no individual patient data or clinical history was recorded, the SERGAS Ethics Committee (the reference Ethics Committee that evaluated the study) determined that the study did not require evaluation or informed consent from patients.

### 2.5 Statistical analysis

The qualitative data obtained in each meeting were processed through a consensus analysis, identifying differences and similarities in the participants' responses. These were transcribed into reports by healthcare area and presented aggregated in the final study report.

Regarding quantitative data, statistical analysis was performed in line with the approved Statistical Analysis Plan, using SAS Enterprise Guide version 7.1 and Microsoft Excel. Continuous variables were described by the number of valid/missing observations, mean, standard deviation (SD), median, 25th and 75th percentiles (P25 and P75, respectively), minimum and maximum were described. Categorical variables were described by frequencies and related percentages. All quantitative data was managed in aggregate form, considering 3 levels of analysis: national level, regional level and by medical specialty.

## 3 Results

### 3.1 Study population

**Figure 1:**
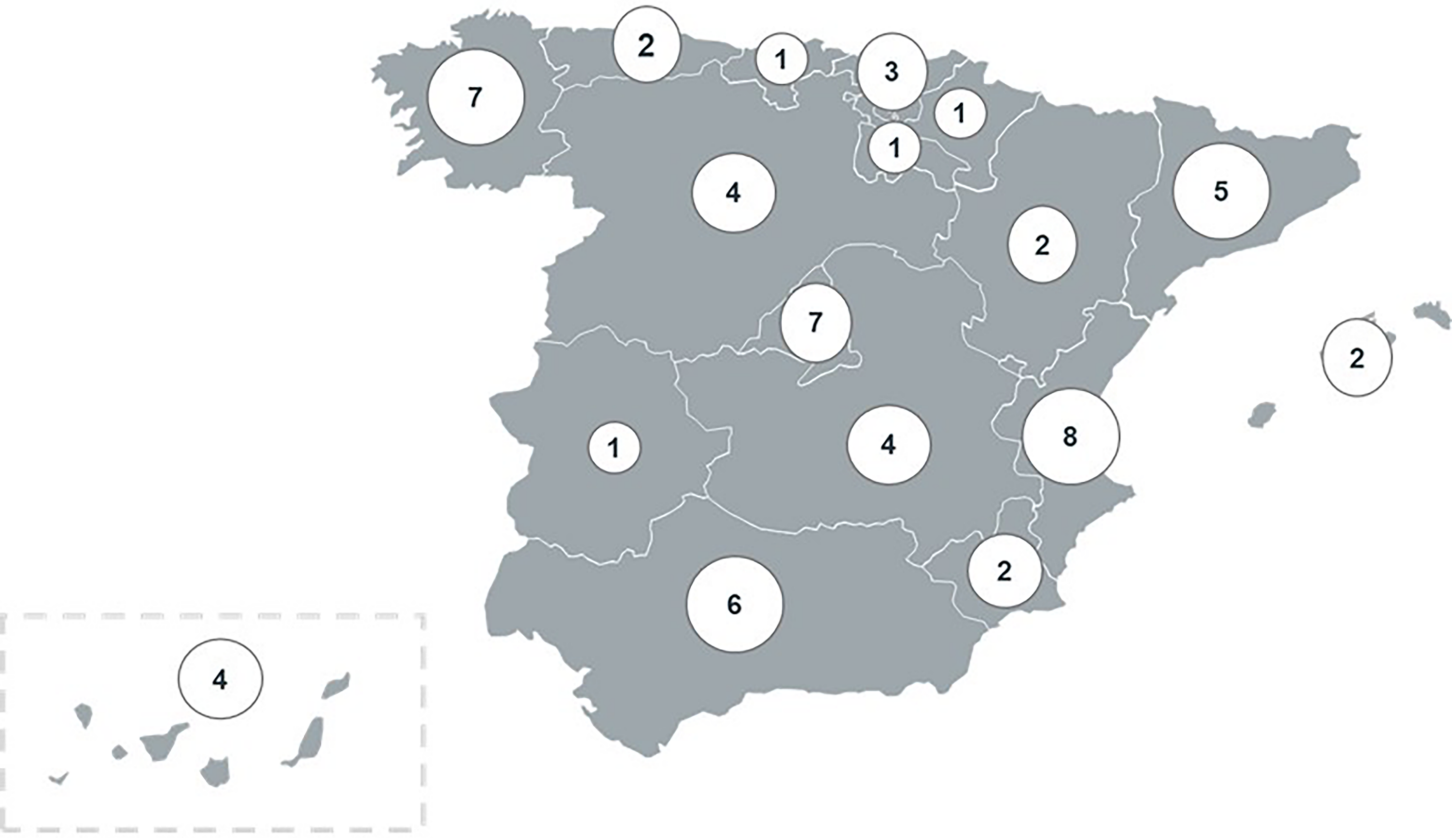
Number of collaborating centers in AMFA Project.

The study involved 180 medical professionals across 60 healthcare areas in Spain (Figure 1). Three experts (one per specialty) by healthcare area were asked to answer a study questionnaire and participate in a multidisciplinary consensus meeting, were answers and further topics were discussed. Of the 180 participants in the AMFA Project, 97.8% completed the questionnaire (*N* = 176) and 98.9% attended the meetings (*N* = 178). Data from 1,580 patients was collected from the 176 experts that completed the questionnaire.

### 3.2 Sociodemographic and clinical characteristics

The mean age (SD) of the 176 experts that completed the questionnaire was 47.9 (±10.3) years, with an average of 21.1 (±10.1) years of clinical experience. Clinical specialty was distributed as follows: 34.7% cardiology, 32.9% general practitioners (GPs), and 32.4% hematology (Table 1). Data of a total of 1,580 patients were collected from expert's clinical practice. In general, the patients exhibited a prevalence of permanent AF of 46.0%. More than half of the patients (55.1%) were over 80 years old and in 83.3% of the cases, body weight was >60 kg. The CHA2DS2-VASc score predominantly ranged between 2 and 4 points (66.8%). The hemorrhagic risk grade values measured using the HASBLED scale were predominantly <3, with 69.3% of patients with a score ≤3. Furthermore, 11.5% of patients had a history of intestinal bleed requiring hospitalization (Table 2).

**Table 1:** Sociodemographic characteristics of physicians participating in the AMFA Project.

Variable	Criteria	Total
Age	Mean (SD)	47.85 (10.34)
	Median	48.00
Clinical experience (years)	Mean (SD)	21.07 (10.12)
	Median	21.50
Specialty	Primary care	58 (32.95%)
	Cardiology	61 (34.66%)
	Hematology	57 (32.39%)

SD, standard deviation.

**Table 2:** Clinical characteristics of patients reported in clinical practice.

Variable	Criteria	Total
AF type	Paroxysmal	533 (33.78%)
	Persistent	319 (20.22%)
	Permanent	726 (46.01%)
Major bleeding risk	HASBLED < 3	1,093 (69.31%)
	HASBLED ≥ 3	484 (30.69%)
CHA 2 DS 2 -VASc score for atrial fibrillation stroke risk	0	22 (1.39%)
	1	176 (11.14%)
	Between 2 and 4	1,056 (66.84%)
	5 or more	326 (20.63%)
Age	>80	710 (44.94%)
	<80	870 (55.06%)
History of intestinal bleed requiring hospitalization	Yes	181 (11.46%)
	No	1,398 (88.54%)

### 3.3 Anticoagulation units

Over 90% of experts reported the presence of anticoagulation units in their healthcare area, primarily located in hospitals (74.1%). Nearly half of these units (46.8%) were considered as reference units (Table 3). In patients starting with a VKA, experts reported that the first INR control occurred at approximately 5 days from prescription for both warfarin and acenocumarol treatments, with scheduled patient follow-up visits occurring every 31 days (Table 4). 40.2% of physicians indicated that they had a specific DOAC's follow-up clinic, mainly depending on hematology department (Table 4), although it is generally used for initial follow-up visits only.

**Table 3:** Presence, location, and recognition of anticoagulation units.

Variable	Criteria	Total
Presence of anticoagulation units for INR control in their healthcare area	Yes (1)	158 (90.80%)
	No	16 (9.20%)
Location of the anticoagulation unit (multiple answer) (1)	Hospital	117 (74.05%)
	PC Center	9 (5.70%)
	PC shared with H	49 (31.01%)
	Other	2 (1.27%)
	In hospital and specialty center	2 (100%)
Anticoagulation unit details (1)	Yes, it is a reference unit	74 (46.84%)
	Yes, but it is not a reference unit	26 (16.46%)
	No	8 (5.06%)
	Don't Know/No Answer	50 (31.65%)

PC, primary care; SD, standard deviation; INR, international normalized ratio; C, cardiology; H, hematology.

**Table 4:** First and follow-up INR control in patients treated with VKAs and presence of specific DOAC clinics reported by specialty.

Variable	Criteria	PC	H	C	Total
Controlled patients INR	Mean (SD)	69.28 (15.75)	63.72 (15.15)	56.07 (15.15)	62.89 (15.37)
	Median	70.00	60.00	60.00	65.00
First INR control for patients treated with warfarin (days)	Mean (SD)	5.41 (4.88)	4.31 (0.93)	5.59 (5.43)	5.04 (4.06)
	Median	4.00	4.50	5.00	4.00
First INR control for patients treated with acenocumarol (days)	Mean (SD)	5.46 (4.60)	3.45 (1.10)	5.12 (3.73)	4.62 (3.55)
	Median	4.00	3.00	4.50	3.00
INR control for stable patients (days)	Mean (SD)	32.79 (17.91)	29.48 (7.27)	30.89 (12.17)	31 (13.28)
	Median	30.00	30.00	30.00	30.00
Specific DOAC Clinic	Yes	10 (17.54%)	34 (62.96%)	24 (41.38%)	68 (40.24%)
	No	47 (82.46%)	20 (37.04%)	34 (58.62%)	101 (59.76%)

DOAC, direct oral anti-coagulant; PC, primary care; C, cardiology; H, hematology; SD, standard deviation; INR, international normalized ratio.

Across each geographic region and individual center, a distinct patient journey unfolded from the initial diagnosis through treatment initiation and subsequent follow-up. This complexity led to the identification of up to 8 patient journey archetypes, i. e. a classification made depending on the healthcare model that each center got. These archetypes help healthcare providers understand and anticipate the diverse ways patients interact with the healthcare system, from initial symptoms to diagnosis, treatment, and follow-up care. Notably, the singular commonality observed across all regions was the diagnosis of AF in PC or emergency departments, with the most complex cases warranting referral to hematology for specialized management.

#### 3.3.1 Prescription of DOACs vs. VKAs

Significant variability was observed in the criteria for initiating DOACs as first-line therapy. It was found that around 56% of the experts prescribed DOACs as first-line treatment under specific conditions, particularly when VKAs were contraindicated and in patients with suboptimal INR control (TTR <60%). Conversely, 39 experts (30.9%) consistently initiated DOACs as first-line therapy, especially cardiologists (47.7%) (Table 5).

**Table 5:** Reported criteria to determine if a patient with AF is a candidate for first-line treatment with DOACs by specialty.

Variable	Criteria	PC	H	C	Total
Criteria to determine if a patient with AF is a candidate for first-line treatment with DOACs (multiple responses)	DOAC as the first option, regardless of criteria	6 (14.29%)	12 (30.00%)	21 (47.73%)	39 (30.95%)
	VKA as the first option, regardless of criteria	12 (28.57%)	6 (15.00%)	1 (2.27%)	19 (15.08%)
	DOAC cannot be prescribed as the first option; a VKA must be started. When prescribing a DOAC, IPT criteria must always be met	26 (61.90%)	22 (55.00%)	22 (50.00%)	70 (55.56%)

DOAC, direct oral anti-coagulant; PC, primary care; C, cardiology; H, hematology; SD, standard deviation; INR, international normalized ratio.

**Figure 2:**
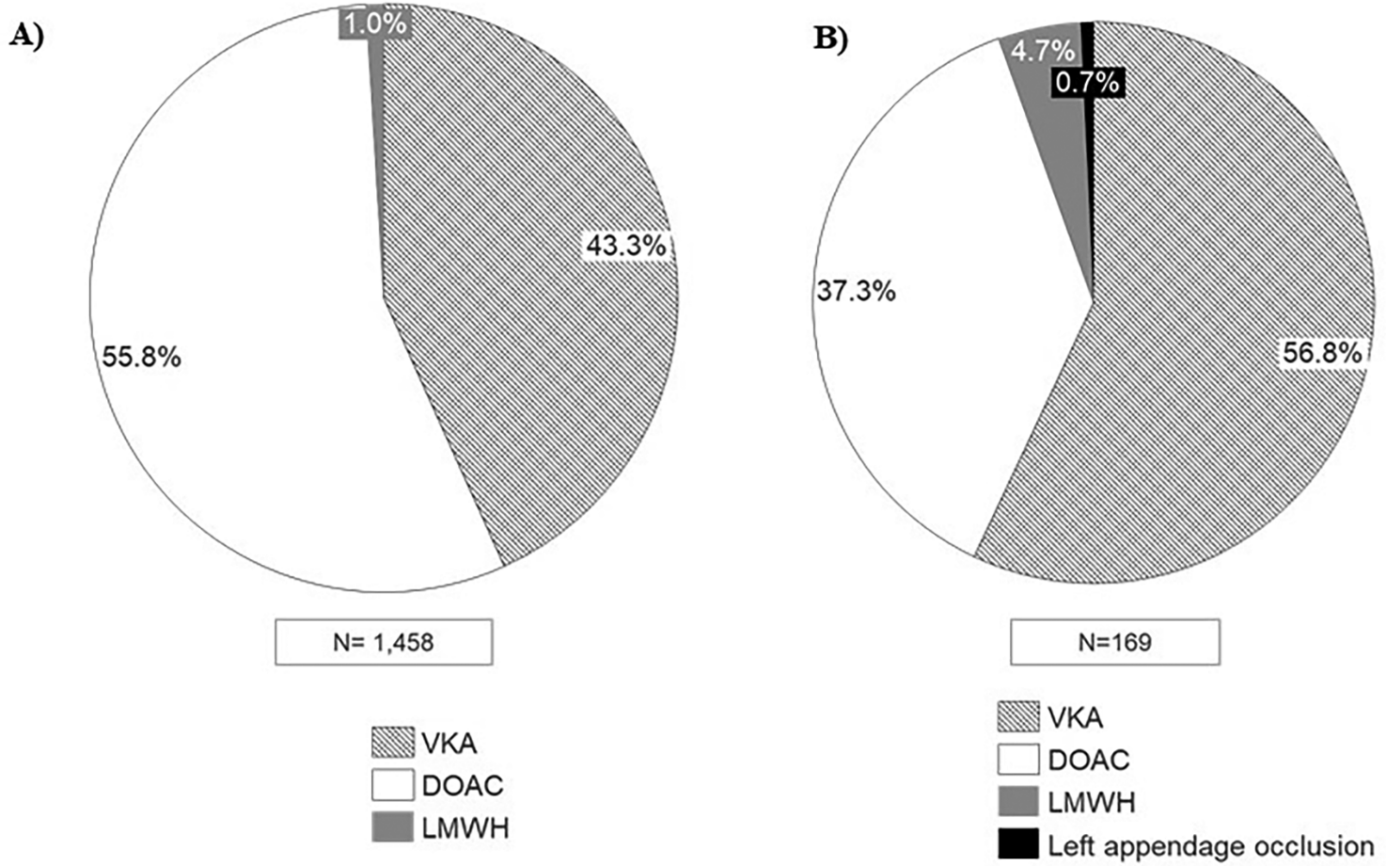
Percentage of patients according to the received treatment. **(A)** Data based on clinical practice patients; **(B)** data based on experts’ perception. VKA, vitamin K antagonists; DOAC, direct oral anti-coagulant; LMWH, low molecular weight heparin.

**Figure 3:**
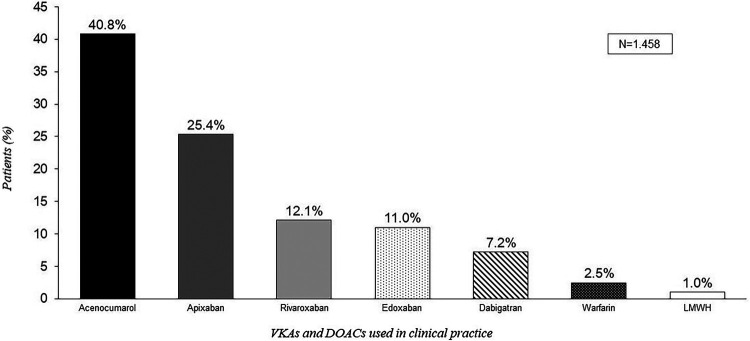
Percentage of patients treated with VKAs and DOACs according to clinical practice data.

According to the results of clinical practice, 43.3% of patients were on treatment with VKAs while 55.8% were on treatment with DOACs (Figure 2B). Acenocumarol was found to be the most frequently prescribed anticoagulant, with 40.8% of treated patients receiving this medication, followed by apixaban in 25.4% of the cases (Figure 3). The use of VKAs and DOACs in clinical practice diverged from the perception of experts, who reported an inverted prescription percentage, with a perception of a higher prescription of VKAs than DOACs (56.8% vs. 37.3%, respectively) (Figure 2A).

#### 3.3.2 Control of coagulation

**Figure 4:**
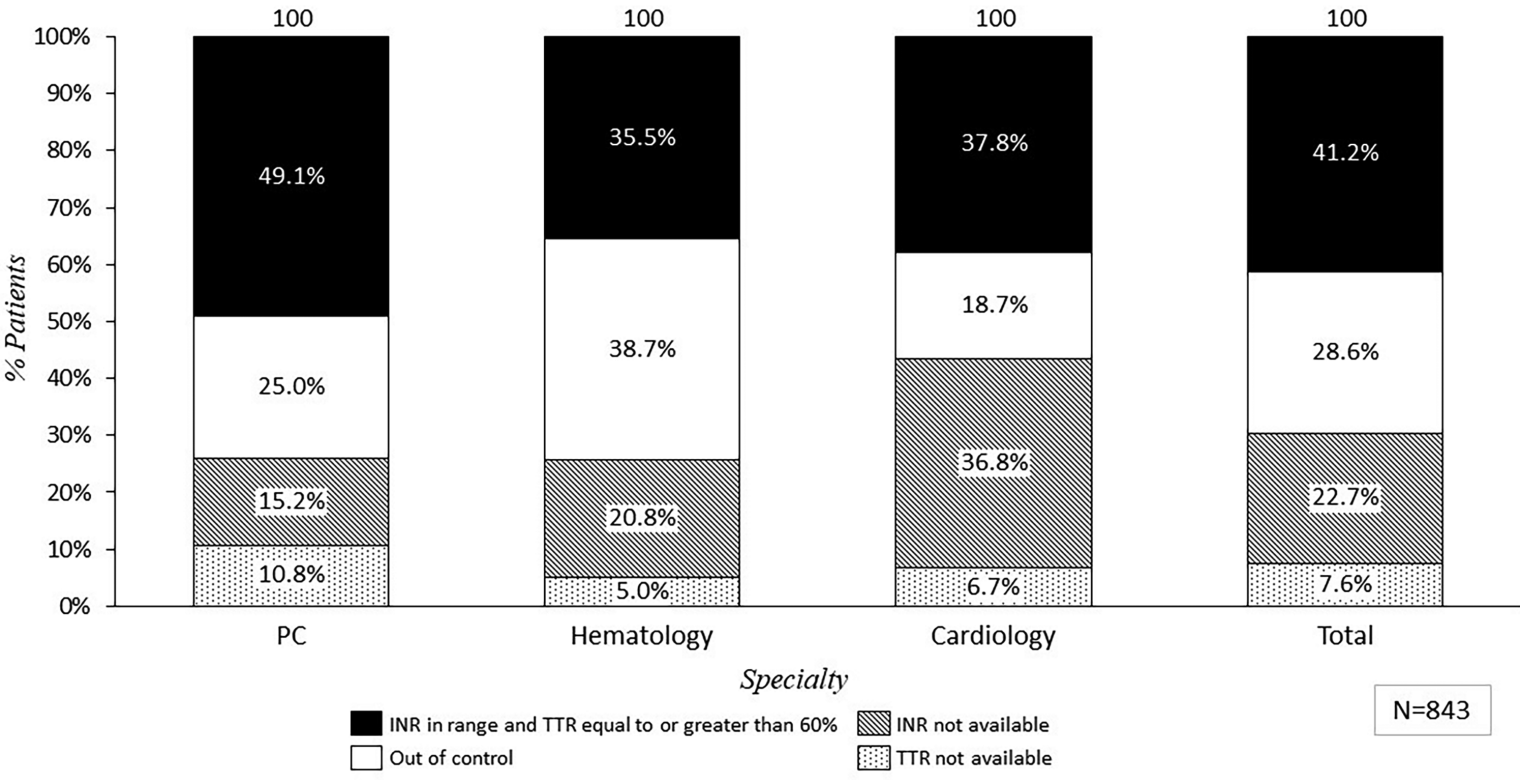
Percentage of patients treated with VKA based on total INR and TRT values reported in clinical practice depending on specialty. PC, primary care; C, cardiology; H, hematology; INR, international normalized ratio; TTR, time in therapeutic range.

Regarding patients' coagulation control, clinical practice revealed that 30.3% of the patients did not have INR nor TTR available in their records (*n_p_* = 255) (Figure 4). Cardiology was the specialty with the higher degree of INR and/or TTR not available (45.5%), followed by hematology and GPs which reported 26.0% and 15.8% of patients without INR and/nor TTR available (Figure 4). This result differs from the experts' perception, since only 3.8% of them claimed that INR/TTR would not be available [0.9% of general practitioners (GP) (±2.7), 3.6% of hematologists (±6.5), and 6.6% of cardiologists (±19.3)].

**Figure 5:**
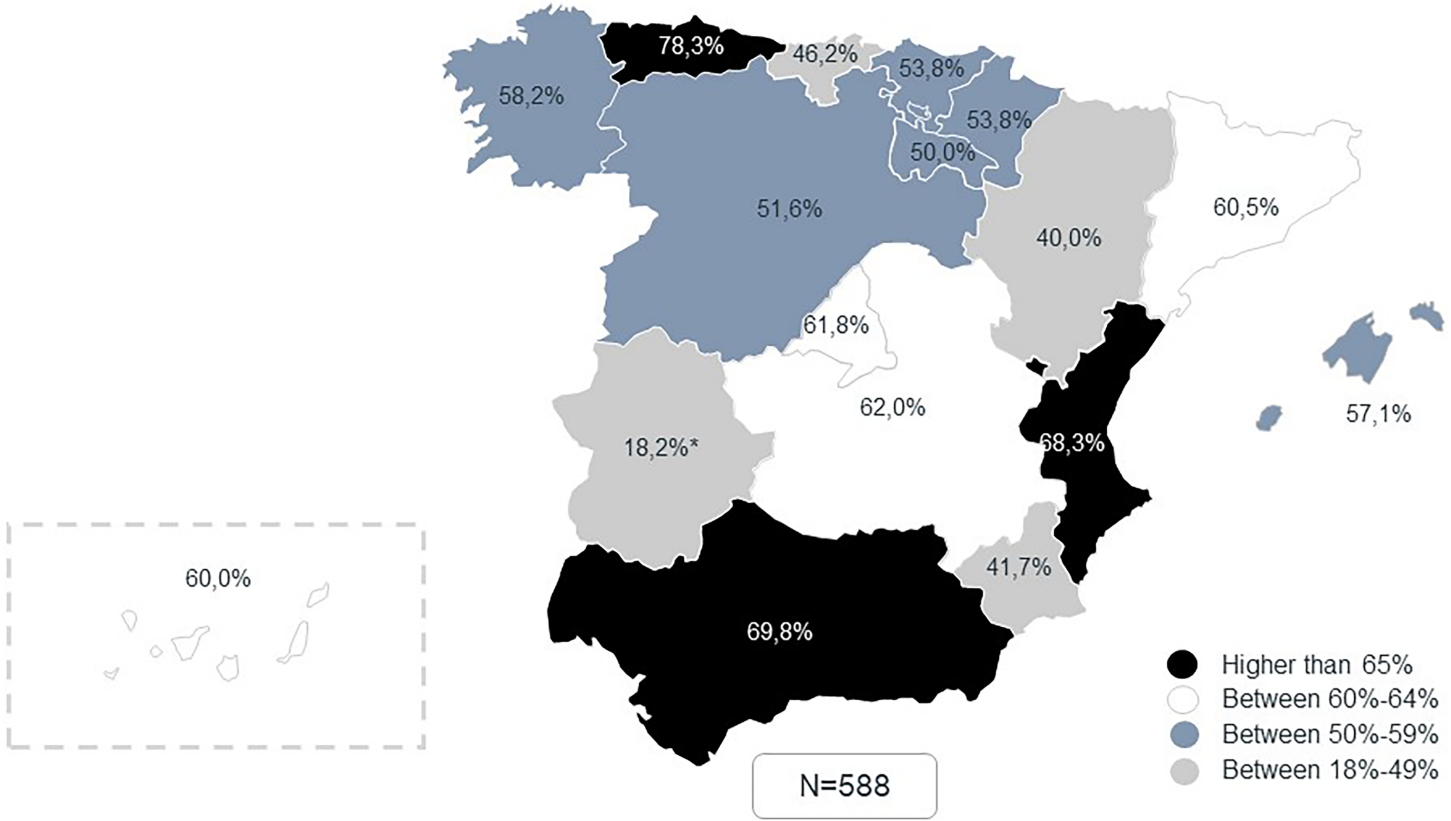
Percentage of patients with adequately controlled coagulation (TTR > 60 0 65%) by region according to clinical practice data. *Extremadura reported only 11 cases, which may bias the results. ^†^Percentages were calculated excluding patients in which INR and/or TRT was not available.

Considering only patients who had INR and TTR available, clinical practice showed that only 59.0% (*n_p_* = 347) of the patients had their anticoagulation treatment in range, while the remaining 41.0% (*n_p_* = 241) were uncontrolled. The distribution of anticoagulation control according to patient data varied across different Spanish regions. As an example, clinical practice from experts in Asturias showed that 78.3% of the patients on VKAs treatment had their coagulation in range, while in Murcia, only 41.7% patients were adequately controlled (Figure 5).

**Figure 6:**
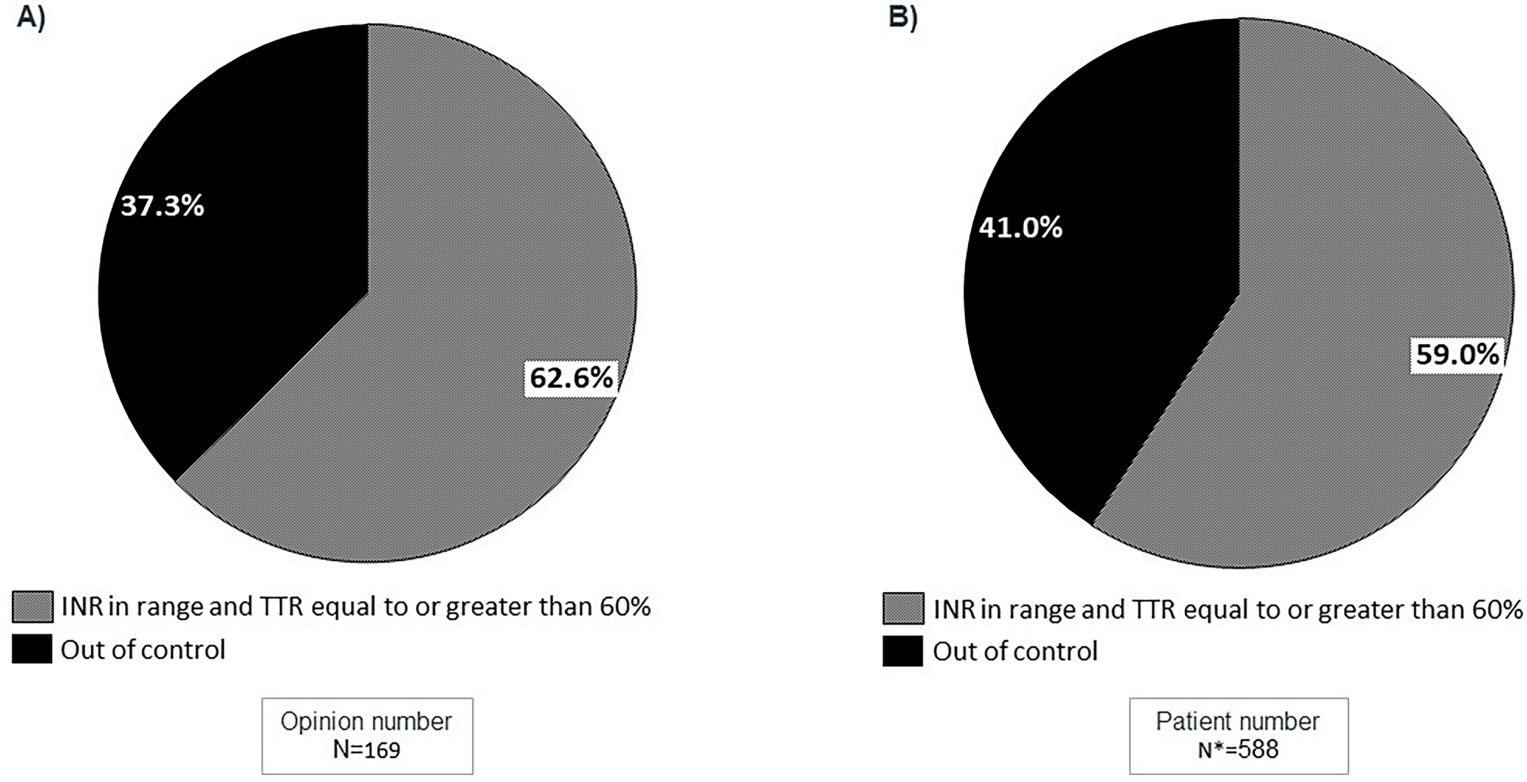
Percentage of patients receiving VKA treatment based on the total INR and TTR values, with the exclusion of patients for whom INR/TTR data is not available. **(A)** Data based on experts’ perception; **(B)** Data based on clinical practice patients. *255 patients in whom INR and/or TRT were not available were excluded of this analysis for comparability reasons.

**Figure 7:**
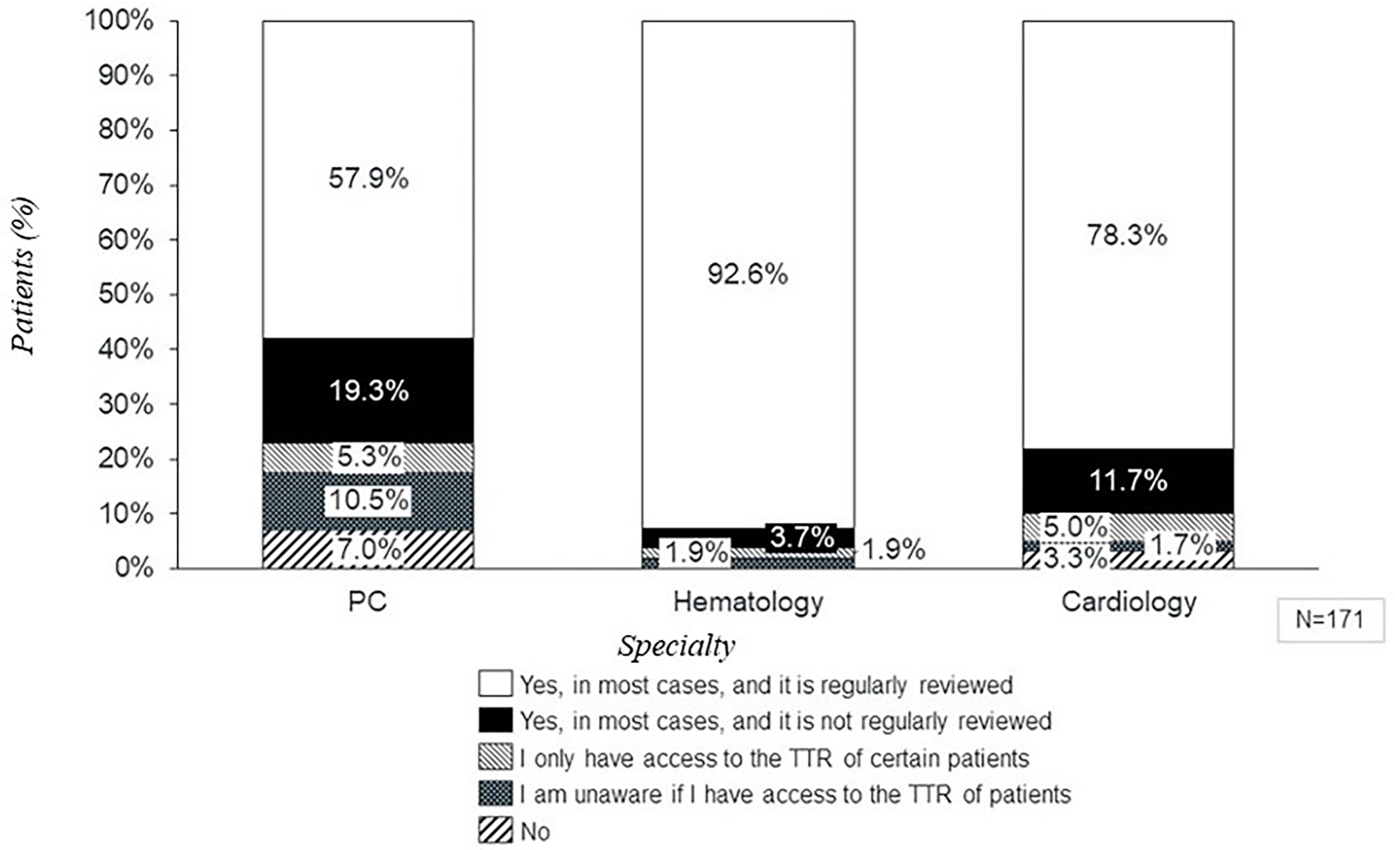
Access to INR and TTR values by specialty according to perception. PC, primary care; C, cardiology; H, hematology; INR, international normalized ratio; TTR, time in therapeutic range.

The mean of appropriated controlled patients (59.0%) was aligned with the general perception reported by the experts, (62.6% of patients in range) (Figure 6). According to perception, GPs reported the lowest rate of reviewing INR and TTR, and 19.3% claimed that although they had access to INR and TTR data, they did not review these data regularly (Figure 7).

**Figure 8:**
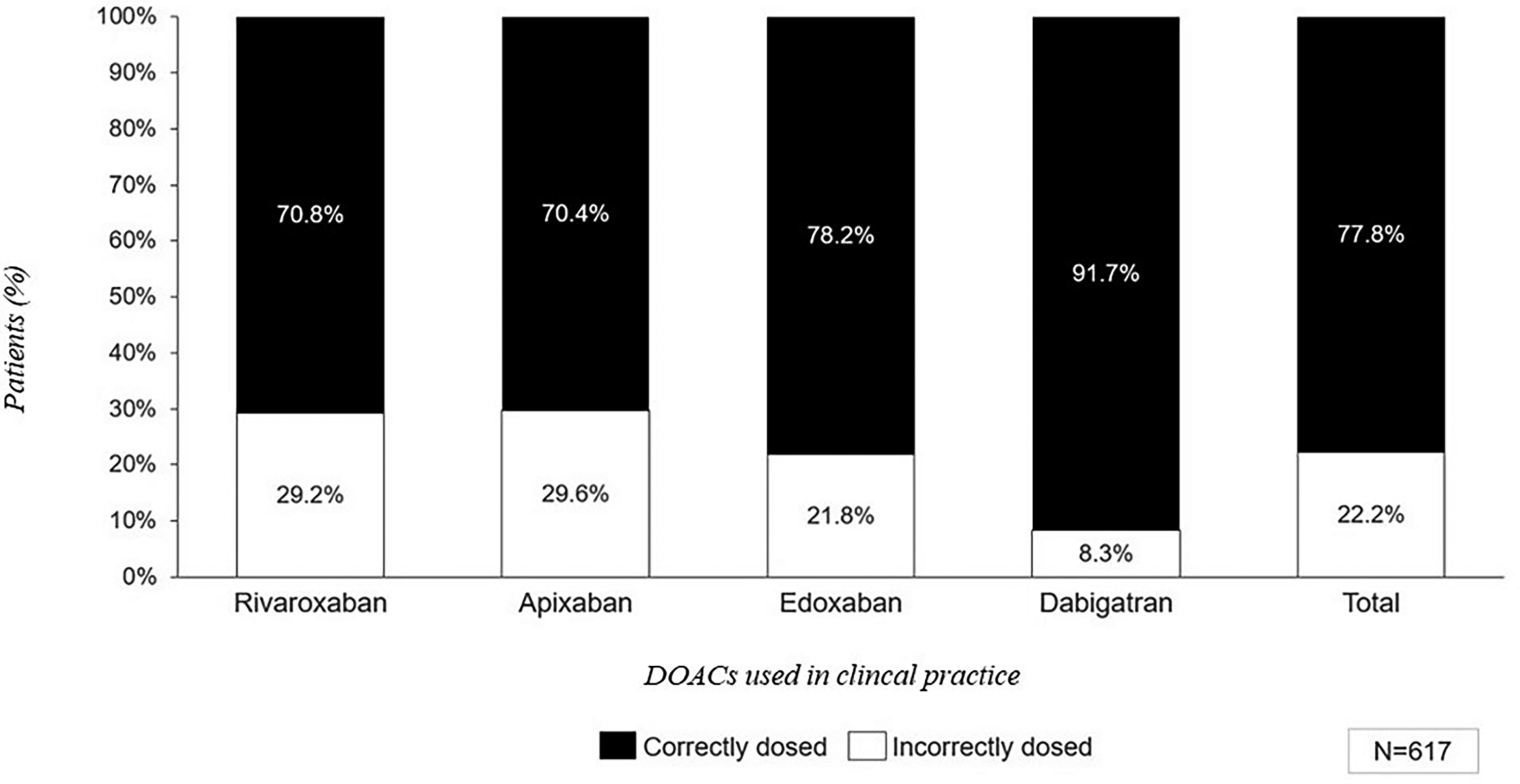
Average number of patients treated with DOACs based on the dosage according to clinical practice data. *Calculations are made based on the summary or product characteristics of each drug, assuming patients receiving edoxaban are not treated concomitantly with P-glycoprotein (P-gp) inhibitors: cyclosporine, dronedarone, erythromycin or ketoconazole.

Regarding DOAC treated patients, clinical practice revealed that up to 22.2% patients received DOACs at inadequate doses (*n_p_* = 617) (Figure 8). Patients treated with dabigatran showed the highest percentage of patients correctly dosed (91.7%), followed by edoxaban (78.2%), rivaroxaban (70.8%), and apixaban (70.4%), respectively. However, expert's perception was more optimistic, reporting a median of only 12.3% (±11.5) of patients with an inappropriate dose of DOACs (Figure 8).

## 4 Discussion

The present study reveals the heterogeneity and suboptimal management of anticoagulation in patients with AF in Spain. As mentioned, effective management of AF involves establishing a proper anticoagulation alongside rhythm and rate control (5). The main pitfalls we found were the high degree of VKA use, the difficulty to access to TTR in many cases and the inadequate dose used in DOACs.

VKAs have been the first line of oral anticoagulant treatment from the early 1950s until recent years. However, its use in clinical practice presents several challenges (20). Currently, clinical guidelines recommend DOACs over VKAs for stroke prevention in AF, due to their greater safety, ease of control, lower degree of drug and food interactions, and the fact that DOACs do not require close monitoring as VKAs (10, 21, 22). However, in Spain, DOACs prescription requires an inspection visa for reimbursement and dispensing at pharmacies (20).

Several previous studies show that Spain lags behind other European countries in the use of DOACs (13). This may result in suboptimal anticoagulation for patients in this country. The findings of the AMFA study have supported this fact. The suboptimal management of anticoagulation in Spain has been attributed to various factors such as inadequate monitoring of both VKAs and DOACs patients, inadequate dosing, lack of consistency across different regions and centers, and challenges related to access and inequality in the access of anticoagulants.

Results of clinical practice have revealed an inadequate monitoring in patients receiving VKA treatment, with 30.3% of doctors reporting that they didn't have access to INR or TTR values for their patients. This is a potential indicator suggesting that patients on VKAs may not be receiving the necessary and frequent monitoring, leading to inadequate control of their anticoagulation treatment. Interestingly, this finding diverges from the perception of clinicians, as only 3.8% of experts claimed during interviews that INR/TTR values were unavailable. These findings could explain why only 59.0% of the analyzed patients had their coagulation within the target range, while the remaining 41.0% were not adequately controlled. Maintaining VKA treatment in patients with poor INR control increases both the risk of thromboembolic and hemorrhagic complications and the associated costs (23).

These results are supported by other studies in the Spanish setting, such as the PAULA study, which showed that only 57%–60% of patients had an adequate INR control (24). The ESPARTA study demonstrated suboptimal control of INR in 57% of patients anticoagulated with VKAs, and also showed non-compliance with the criteria for the use of DOACs issued by the Spanish Agency of Medicines and Medical Devices (AEMPS) (25).

Despite the good correlation between perceived and actual TTR, many physicians still considered DOACs as a second-line therapy compared to VKAs. This preference is due to several factors: (a) Historical preference and familiarity: VKAs have been the standard for many decades, leading to greater familiarity among physicians; (b) Regulatory and reimbursement policies: In Spain, prescribing DOACs often requires an inspection visa for reimbursement, creating administrative hurdles; (c) Clinical guidelines and protocols: Some guidelines and protocols still prioritize VKAs as the initial treatment; (d) Perceived cost and accessibility: DOACs are more expensive than VKAs, influencing decisions in budget-constrained healthcare systems; and (e) Patient-specific factors: Individual patient characteristics, such as comorbidities and preferences, can make VKAs more suitable in certain cases. Addressing these barriers is important to promote the appropriate use of DOACs where clinically indicated.

In AMFA study, the 90.80% of the specialists reported that their center did have a specific anticoagulation unit. These results are interesting in the context of the suboptimal control of anticoagulation in Spain reported by the AMFA study. The presence of specific anticoagulation units would lead to think about better control of anticoagulation. However, the results of the AMFA study demonstrate that there is no direct relation between the presence of anticoagulation units and better control of anticoagulation. These data reveal that there are other factors involved in the control of anticoagulation that hinder or do not facilitate optimal control of anticoagulation, which open a possible area of ​​research to elucidate the reasons why in Spain and, despite having the necessary tools, the control of VKA anticoagulated patients is not being carried out adequately.

It is important to note that the inadequate of control described in the AMFA study is not exclusive to VKAs. Clinical practice results indicate that a significant number of patients receiving DOAC treatment were prescribed either higher or lower doses than the recommended; these patients corresponded to 22.2%. Specifically, different DOACs showed similar patterns, although edoxaban could be slightly overestimated because of the assumption that patients were not treated with P-glycoprotein inhibitors. In addition, low dose of dabigatran is considered always as “correct”, since there are some guidelines that recommend it, explaining the high degree of correctly dosed patients. The data confronted clinicians' perceptions, as it was reported that a median of 12.3% of patients received inappropriate doses of DOACs, underlining the lack of awareness of this issue in clinical practice setting. In this context, the Spanish FANTASIIA Registry identified 32% of patients anticoagulated with DOACs receiving inappropriate doses, leading to worse health outcomes when compared to those correctly anticoagulated (26). Similarly, a study published in 2023 revealed that nearly one-fourth of patients over 75 years old treated with DOACs in Spain were inappropriately dosed (27).

It is also important to highlight that it was reported that 0.7% patients received left appendage occlusion, evidencing that this procedure is recognized for its effectiveness in managing atrial fibrillation, but its use is typically guided by patient-specific factors, including the severity of the condition, the presence of other health issues, and the patient's overall risk profile (28). The left appendage occlusion is considered a reasonable option for these patients, but it is not the first-line treatment according to the American College of Cardiology (29), aligned with our results. According to study, the most common procedure is the left appendage occlusion for patients with non-valvular atrial fibrillation (28). However, other procedures used in these patients, although not studied in our study, are Cox-Maze procedures for controlling atrial fibrillation with limited data of prevalence in Spain.

An additional factor that could be contributing to the lack of anticoagulation control across the Spanish territory is the significant heterogeneity in the management of anticoagulation across different healthcare centers. Each geographic region and individual center demonstrated a unique patient flow from AF diagnosis through treatment initiation and subsequent follow-up. This diversity led to the identification of up to 8 distinct patient journey archetypes, underscoring the intricate and multifaceted nature of anticoagulation treatment in Spain. These results showcased the highly variable clinical practices observed, highlighting the substantial impact of geographical location and even the specific hospital where patients receive care. Consequently, it is apparent that the management of anticoagulation treatment in Spain is influenced by a multitude of factors, resulting in a complex and diverse landscape of clinical practices across the country.

Multiple studies conducted in several European countries have shown that effective anticoagulation control results in a decrease in cardiac events and stroke (30, 31). In addition, the introduction of DOACs has also demonstrated an improvement in patient outcomes in the healthcare setting of European countries (32, 33). Similarly, a population-based epidemiological study conducted in Spain from 2005 to 2018 indicated that prior to the introduction of DOACs, the incidence rate of AF-related ischemic stroke experienced a consistent rise from 2005 to 2012 (20). However, subsequent to the adoption of DOACs in Spain for the prevention of AF-related ischemic stroke from 2012 onwards, the incidence rate remained stable or showed a slight decrease through 2018 (20).

Although the AMFA study has not studied the economic impact of DOACs vs. VKAs or the side effects of optimal anticoagulation, based on the literature, it seems evident that promoting and expanding the use of DOACs would be beneficial in social, health and economic terms.

Besides, our study did not specifically analyze the age of the physicians in relation to their prescribing habits. However, we did observe some regional variations in the use of DOACs vs. VKAs. These variations can be influenced by several factors, including regional healthcare policies, availability of resources, and local clinical guidelines.

Study's main strength lies in the inclusion of a high number of specialists representing all regions in Spain and providing a comprehensive perspective on anticoagulant treatment management.

This study also showed comparisons between physicians' perceptions and real-world patient outcomes, offering a valuable contribution to the understanding of anticoagulant therapy in Spain. The main limitations of the study were the incorporation of aggregated clinical practice data, as well as the amount of missing data.

The AMFA study uncovers inadequate INR access and control in patients on VKA treatment and highlights regional disparities. It also brings to light the gap the discrepancies between perceived and actual anticoagulation status in Spain. This emphasizes the need to implement strategies to improve INR control, enhance access to DOACs, and standardize AF patient management through the establishment of protocols for optimized care.

To optimize anticoagulation management in Spain, it is crucial to increase the use of DOACs and improve INR control. This involves better identification of patients with uncontrolled INR, as well as enhancing training and communication among various medical specialties. However, it is necessary to implement different strategies and improvements to achieve this objective. Furthermore, the study recommends the integration of advanced technologies, such as telemedicine and electronic health records, to facilitate better monitoring and management of anticoagulation therapy. Collaboration between healthcare providers, policymakers, and patient advocacy groups is also emphasized as a key factor in driving these improvements.

## Data Availability

The raw data supporting the conclusions of this article will be made available by the authors, without undue reservation.
